# Supplementary material for: Seven Fatty Acid Metabolism-Related Genes as Potential Biomarkers for Predicting the Prognosis and Immunotherapy Responses in Patients with Esophageal Cancer
Source: Vaccines (Basel). 2022 Oct 15;10(10):1721. doi: 10.3390/vaccines10101721 (PMC9610070; doi:10.3390/vaccines10101721)
Supplement: Supplementary file 1 [file vaccines-10-01721-s001.zip › Supplementary Table S1.pdf]

Table S1 Details of clinical characteristics

Id	Age	Gender	Stage	T	N	M
TCGA-2H-A9GJ	57	MALE	Stage I	T1	N0	M0
TCGA-L5-A891	51	MALE	unknow	T3	N1	M0
TCGA-L5-A43I	unknow	MALE	Stage IIIA	T3	N1	MX
TCGA-LN-A49K	unknow	MALE	Stage IIA	T3	N0	M0
TCGA-LN-A49S	59	MALE	Stage IIA	T3	N0	M0
TCGA-IG-A8O2	62	MALE	Stage IIIB	T3	N2	M0
TCGA-S8-A6BW	51	MALE	Stage IB	T2	N0	MX
TCGA-IG-A3I8	51	FEMALE	Stage IIA	T3	N0	M0
TCGA-LN-A49V	unknow	MALE	Stage IIA	T3	N0	M0
TCGA-Q9-A6FW	61	MALE	Stage IIIB	T3	N2	M0
TCGA-L5-A4OF	63	MALE	Stage IIB	T1	N1	unknow
TCGA-IG-A625	60	MALE	Stage IIIB	T3	N2	M0
TCGA-LN-A4A1	60	MALE	Stage IIA	T3	N0	M0
TCGA-JY-A6FB	77	MALE	Stage I	T1	N0	M0
TCGA-LN-A49M	62	MALE	Stage IIA	T2	N0	M0
TCGA-R6-A6Y2	unknow	MALE	unknow	unknow	unknow	unknow
TCGA-LN-A7HV	58	MALE	Stage IIA	T2	N0	M0
TCGA-LN-A49W	73	MALE	Stage III	T3	N1	M0
TCGA-L5-A43C	81	MALE	unknow	unknow	unknow	unknow
TCGA-LN-A49N	unknow	MALE	Stage IIB	T2	N1	M0
TCGA-LN-A7HW	59	MALE	Stage IIA	T2	N0	M0
TCGA-2H-A9GQ	80	MALE	Stage III	T3	N1	M0
TCGA-JY-A93E	61	MALE	Stage IIIA	T3	N1	M0
TCGA-VR-A8EO	49	MALE	Stage IIA	T3	N0	M0
TCGA-LN-A4A6	unknow	MALE	Stage II	T2	N0	M0
TCGA-L5-A88Z	70	FEMALE	Stage IIA	T1	N1	M0
TCGA-VR-A8EX	63	MALE	Stage IVA	T1	N1	M1a
TCGA-R6-A8W8	72	MALE	unknow	unknow	unknow	unknow
TCGA-L5-A893	71	FEMALE	Stage I	T1	N0	M0
TCGA-R6-A6XQ	58	MALE	unknow	unknow	unknow	unknow
TCGA-Z6-AAPN	57	MALE	Stage IIA	T3	N0	M0
TCGA-LN-A49O	47	MALE	Stage IIA	T3	N0	M0
TCGA-X8-AAAR	unknow	MALE	unknow	T1	N1	unknow
TCGA-IG-A3Y9	unknow	MALE	Stage IIIA	T4	N0	M0
TCGA-IG-A6QS	54	MALE	Stage IIB	T2	N1	M0
TCGA-S8-A6BV	76	MALE	Stage IIIA	T3	N1	MX
TCGA-L5-A8NI	79	MALE	Stage III	T3	N1	M0
TCGA-R6-A6DQ	74	FEMALE	unknow	unknow	unknow	unknow
TCGA-JY-A93C	47	MALE	Stage IIIB	T3	N2	M0
TCGA-RE-A7BO	72	FEMALE	Stage IIB	T1	N1	M0

TCGA-VR-A8EY	44	FEMALE	Stage IIA	T3	N0	M0
TCGA-2H-A9GL	74	MALE	Stage III	T3	N1	M0
TCGA-L5-A88S	84	MALE	Stage IB	T3	N0	MX
TCGA-LN-A5U7	46	MALE	Stage IIA	T2	N0	M0
TCGA-XP-A8T6	54	MALE	Stage IIB	T2	N1	M0
TCGA-IG-A4P3	48	MALE	Stage IIB	T2	N1	M0
TCGA-R6-A6XG	64	MALE	unknow	unknow	unknow	unknow
TCGA-L5-A43H	unknow	MALE	Stage III	T3	N1	M0
TCGA-LN-A9FP	60	FEMALE	Stage IIA	T2	N0	M0
TCGA-KH-A6WC	82	MALE	Stage IA	T1	N0	M0
TCGA-JY-A6FE	49	MALE	Stage III	T3	N1	M0
TCGA-R6-A6Y0	54	MALE	unknow	unknow	unknow	unknow
TCGA-Z6-A9VB	53	MALE	Stage IIIA	T3	N1	M0
TCGA-IG-A4QT	unknow	MALE	Stage IIA	T3	N0	M0
TCGA-L5-A8NM	84	FEMALE	Stage IIB	T2	N1	M0
TCGA-L5-A4OW	56	FEMALE	Stage IIB	T2	N1	M0
TCGA-LN-A4MR	unknow	MALE	Stage IIA	T2	N0	M0
TCGA-VR-A8Q7	60	MALE	Stage IIIA	T3	N1	M0
TCGA-IG-A5B8	72	MALE	Stage IB	T3	N0	M0
TCGA-LN-A4A3	61	MALE	Stage III	T3	N1	M0
TCGA-LN-A49X	44	MALE	Stage IIA	T3	N0	M0
TCGA-L5-A4OQ	75	MALE	Stage IIIA	T2	N2	MX
TCGA-VR-AA4G	51	FEMALE	Stage IIIA	T2	N2	M0
TCGA-R6-A6L6	unknow	MALE	unknow	unknow	unknow	unknow
TCGA-IG-A51D	63	MALE	Stage IIB	T1	N1	M0
TCGA-L5-A4OE	81	MALE	Stage IIIB	T3	N2	unknow
TCGA-VR-AA7B	unknow	FEMALE	Stage IV	T3	N3	M1
TCGA-LN-A4A8	52	MALE	Stage IIA	T2	N0	M0
TCGA-LN-A9FO	42	MALE	Stage IIA	T2	N0	M0
TCGA-R6-A8WC	56	MALE	unknow	unknow	unknow	unknow
TCGA-IC-A6RE	59	MALE	Stage IIB	T1	N1	M0
TCGA-VR-A8ER	54	MALE	Stage III	T4	N1	M0
TCGA-JY-A6FH	53	MALE	Stage IIB	T2	N1	M0
TCGA-JY-A6F8	unknow	MALE	Stage I	T1	N0	M0
TCGA-XP-A8T8	49	MALE	Stage IIB	T1	N1	M0
TCGA-L5-A8NN	81	MALE	Stage III	T3	N1	M0
TCGA-LN-A9FR	70	MALE	Stage IIB	T2	N1	M0
TCGA-L5-A88Y	76	MALE	unknow	T1	N0	MX
TCGA-L5-A8NV	75	MALE	Stage IIA	T3	N0	M0
TCGA-L5-A8NL	56	MALE	Stage III	T3	N1	M0
TCGA-IG-A7DP	50	FEMALE	Stage IIIA	T4a	NX	MX
TCGA-LN-A7HZ	49	MALE	Stage IIA	T2	N0	M0
TCGA-IG-A3YA	53	MALE	Stage IIIA	T4	N0	M0
TCGA-L5-A4OU	81	MALE	Stage IIA	T3	N0	M0

TCGA-JY-A6FA	51	MALE	Stage IIB	T2	N1	M0
TCGA-JY-A93D	51	MALE	Stage IIIC	T2	N3	M0
TCGA-L5-A8NR	81	FEMALE	Stage III	T3	N1	M0
TCGA-LN-A5U5	57	MALE	Stage IV	T3	N1	M1
TCGA-L5-A43E	74	MALE	Stage I	T1	N0	M0
TCGA-L7-A6VZ	62	MALE	Stage IIIC	T3	N3	MX
TCGA-VR-AA7I	70	MALE	Stage III	T4	N0	M0
TCGA-2H-A9GR	80	MALE	Stage IIA	T3	N0	M0
TCGA-VR-AA7D	unknow	MALE	Stage IIIC	T3	N3	M0
TCGA-Z6-A8JD	53	MALE	Stage IIB	T3	N0	M0
TCGA-L5-A8NQ	71	MALE	Stage IIA	T2	N0	M0
TCGA-V5-A7RB	59	MALE	unknow	T0	N1	MX
TCGA-LN-A4A2	unknow	MALE	Stage IIA	T3	N0	M0
TCGA-LN-A49U	62	MALE	Stage IIA	T3	N0	M0
TCGA-2H-A9GH	44	MALE	Stage IIB	T1	N1	M0
TCGA-IG-A97H	36	MALE	Stage IIA	T3	NX	M0
TCGA-JY-A939	77	MALE	Stage IIB	T1	N1	M0
TCGA-R6-A8W5	60	MALE	Stage IVA	T3	N1	M1a
TCGA-IG-A3YC	62	MALE	Stage IIIA	T3	N1	M0
TCGA-IG-A3QL	54	MALE	Stage IIA	T2	N0	M0
TCGA-M9-A5M8	58	MALE	Stage IIA	T3	N0	M0
TCGA-L5-A4OG	79	FEMALE	Stage I	T1	N0	M0
TCGA-L5-A4OR	83	MALE	Stage IA	T1	N0	MX
TCGA-Z6-A8JE	57	MALE	Stage IIIA	T3	N1	M0
TCGA-R6-A6KZ	42	MALE	unknow	unknow	unknow	unknow
TCGA-LN-A49L	unknow	MALE	Stage IIA	T2	N0	M0
TCGA-R6-A6DN	58	MALE	unknow	unknow	unknow	unknow
TCGA-R6-A6L4	27	MALE	unknow	unknow	unknow	unknow
TCGA-LN-A4MQ	46	MALE	Stage III	T3	N1	M0
TCGA-VR-A8EP	51	MALE	Stage IIIB	T3	N2	M0
TCGA-L5-A4OH	71	MALE	Stage I	T1	N0	M0
TCGA-L5-A8NE	77	MALE	Stage IIB	T2	N1	M0
TCGA-LN-A4A5	49	MALE	Stage IIA	T2	N0	M0
TCGA-L5-A4ON	65	MALE	Stage IIB	T1	N1	unknow
TCGA-L5-A8NT	69	MALE	Stage IIB	T3	N0	M0
TCGA-L5-A4OT	77	MALE	Stage IV	T3	N1	M1a
TCGA-L5-A8NH	54	MALE	Stage IV	T1	N0	M1
TCGA-2H-A9GO	58	MALE	Stage IVA	T3	N1	M1a
TCGA-2H-A9GN	70	MALE	Stage III	T3	N1	M0
TCGA-L5-A4OP	67	FEMALE	Stage IA	T1	N0	MX
TCGA-IG-A3YB	61	MALE	Stage IIIA	T3	N1	M0
TCGA-LN-A8I1	67	FEMALE	Stage IIA	T2	N0	M0
TCGA-LN-A5U6	54	MALE	Stage IIB	T2	N1	M0
TCGA-LN-A8HZ	56	MALE	Stage IIA	T2	N0	M0

TCGA-VR-A8ET	64	MALE	Stage IIA	T2	N0	M0
TCGA-2H-A9GG	66	MALE	Stage III	T3	N1	M0
TCGA-L5-A4OI	79	MALE	Stage IIIC	T3	N3	MX
TCGA-L5-A88T	86	MALE	Stage IIB	T2	N1	M0
TCGA-2H-A9GF	67	MALE	Stage III	T3	N1	M0
TCGA-L5-A8NW	55	MALE	unknow	T2	N1	M0
TCGA-VR-A8EZ	47	MALE	Stage IIIC	T3	N3	M0
TCGA-L5-A4OM	54	FEMALE	Stage IA	T1	N0	unknow
TCGA-2H-A9GI	68	MALE	Stage III	T3	N1	M0
TCGA-L5-A8NF	57	MALE	Stage IVA	T1	N0	M1a
TCGA-V5-A7RC	55	MALE	unknow	unknow	unknow	unknow
TCGA-L5-A8NU	84	MALE	Stage IIA	T2	N0	M0
TCGA-JY-A6FD	51	FEMALE	Stage IIA	T3	N0	M0
TCGA-V5-A7RE	45	MALE	Stage IB	T1	N0	M0
TCGA-ZR-A9CJ	65	MALE	Stage IIIC	T3	N3	MX
TCGA-L5-A43M	unknow	FEMALE	unknow	T3	N2	MX
TCGA-V5-AASX	74	MALE	unknow	unknow	unknow	unknow
TCGA-LN-A49P	71	MALE	Stage IIA	T3	N0	M0
TCGA-LN-A8I0	52	MALE	Stage IIA	T2	N0	M0
TCGA-VR-A8EW	57	MALE	Stage IIIB	T3	N2	M0
TCGA-L5-A43J	90	MALE	Stage IIB	T3	N0	MX
TCGA-IC-A6RF	69	FEMALE	Stage IA	T1	N0	M0
TCGA-LN-A49R	unknow	MALE	Stage III	T3	N1	M0
TCGA-2H-A9GM	53	MALE	Stage IIB	T1	N1	M0
TCGA-LN-A7HX	72	MALE	Stage IIA	T2	N0	M0
TCGA-2H-A9GK	43	MALE	Stage III	T3	N1	M0
TCGA-V5-AASV	67	MALE	Stage IIB	T3	N0	MX
TCGA-IG-A4QS	71	MALE	Stage IIIB	T3	N2	M0
TCGA-L5-A8NG	77	MALE	Stage III	T3	N1	M0
TCGA-VR-A8EU	51	MALE	Stage IV	T1	N1	M1
TCGA-VR-A8EQ	73	MALE	Stage III	T3	N1	M0
TCGA-L5-A4OO	75	MALE	Stage IIIC	T3	N3	M0
TCGA-R6-A8WG	60	MALE	unknow	unknow	unknow	unknow
TCGA-IG-A50L	58	MALE	Stage IIIA	T3	N1	M0
TCGA-L5-A4OJ	70	FEMALE	Stage I	T1	N0	M0
TCGA-V5-AASW	72	MALE	unknow	unknow	unknow	unknow
TCGA-L5-A8NS	76	MALE	Stage IIB	T3	N0	M0
TCGA-LN-A4A9	58	MALE	Stage IIA	T2	N0	M0
TCGA-L5-A8NK	84	FEMALE	Stage IIA	T3	N0	M0
TCGA-L5-A88W	67	MALE	Stage IIA	T3	N0	MX
TCGA-IG-A5S3	69	FEMALE	Stage IIB	T3	N0	M0
TCGA-Q9-A6FU	unknow	FEMALE	Stage IIIB	T3	N2	M0
TCGA-L5-A88V	60	MALE	Stage III	T3	N1	MX
TCGA-JY-A93F	58	FEMALE	Stage IB	T2	N0	M0

TCGA-IG-A97I	58	MALE	Stage IIA	T2	N0	M0
TCGA-L5-A4OS	86	FEMALE	Stage IIB	T2	N1	M0
TCGA-XP-A8T7	unknow	FEMALE	Stage IIA	T2	N0	M0
TCGA-L7-A56G	65	MALE	unknow	unknow	unknow	unknow
TCGA-L5-A4OX	60	MALE	Stage IIB	T2	N1	M0
TCGA-L5-A8NJ	77	MALE	Stage III	T3	N1	M0
TCGA-LN-A49Y	77	MALE	Stage IIA	T3	N0	M0
TCGA-VR-AA4D	53	MALE	Stage IIB	T1	N1	M0
TCGA-LN-A4A4	36	MALE	Stage III	T3	N1	M0
TCGA-LN-A9FQ	62	MALE	Stage IIA	T3	N0	M0
TCGA-LN-A7HY	50	MALE	Stage III	T3	N1	M0
