# Supplementary material for: Seven Fatty Acid Metabolism-Related Genes as Potential Biomarkers for Predicting the Prognosis and Immunotherapy Responses in Patients with Esophageal Cancer
Source: Vaccines (Basel). 2022 Oct 15;10(10):1721. doi: 10.3390/vaccines10101721 (PMC9610070; doi:10.3390/vaccines10101721)
Supplement: Supplementary file 1 [file vaccines-10-01721-s001.zip › Supplementary Table S2.pdf]

Table S2 Summarized clinical information

Clinical feature	Type	Numbers of patients
Age	<=65	99
	>65	63
	unknow	21
Gender	Male	156
	Female	27
Stage I	Stage I	18
	Stage II	78
	Stage III	55
	Stage IV	9
	unknow	23
T	T0	1
	T1	31
	T2	43
	T3	86
	T4	5
	unknow	17
N	N0	76
	N1	68
	N2	12
	N3	8
	unknow	19
M	M0	134
	M1	9
	unknow	40
