# Supplementary figures and images for: Seven Fatty Acid Metabolism-Related Genes as Potential Biomarkers for Predicting the Prognosis and Immunotherapy Responses in Patients with Esophageal Cancer
Source: Vaccines (Basel). 2022 Oct 15;10(10):1721. doi: 10.3390/vaccines10101721 (PMC9610070; doi:10.3390/vaccines10101721)

A

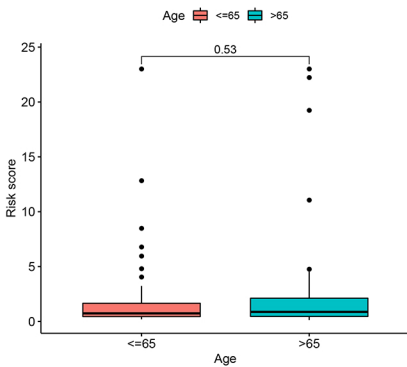

B

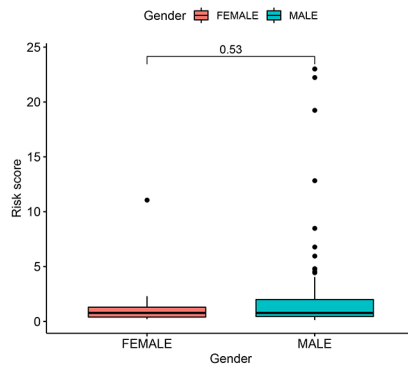

C

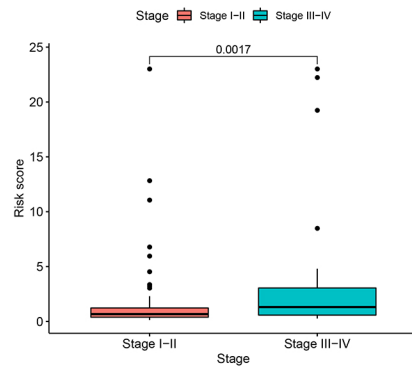

D

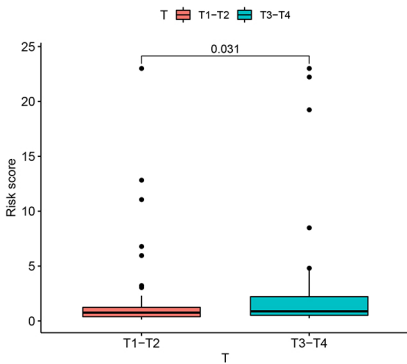

E

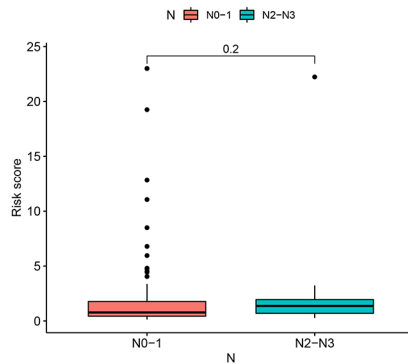

F

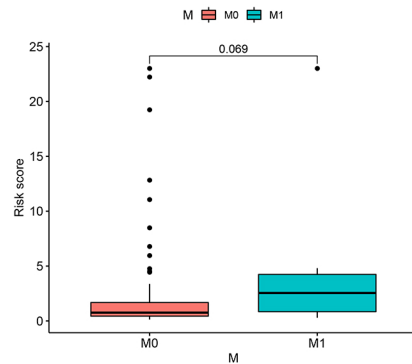

The association between risk score and clinical characteristics

Supplement: Supplementary file 1 [file vaccines-10-01721-s001.zip › Figure S1.pdf]
